# Supplementary material for: Fine mapping and candidate gene analysis of Dravet syndrome modifier loci on mouse chromosomes 7 and 8
Source: Mamm Genome. 2024 Jun 11;35(3):334–45. doi: 10.1007/s00335-024-10046-3 (PMC11329421; doi:10.1007/s00335-024-10046-3)
Supplement: Supplementary file 1 — (PDF 65 KB) [file 335_2024_10046_MOESM1_ESM.pdf]

Supplementary Table S1. Summary of 129S6/SvEvTac versus C57BL/6J variants in *Dsm2b* and *Dsm3* Intervals

	Dsm2b		***Dsm3***	
**Functional Category**	**SNPs**	**Indels**	**SNPs**	**Indels**
Intergenic region	5015	1594	1008	392
Intron variant	3893	1397	179	149
Downstream gene variant	66	22	-	4
Upstream gene variant	41	29	-	-
3’ UTR variant	22	17	-	2
5’ UTR variant	4	-	-	-
Synonymous variant	29	-	2	-
Missense variant	10	-	3	-
Splice region variant	4	1	-	-
Disruptive inframe deletion	-	1	-	-
Inframe insertion	-	1	-	-
TOTAL	9084	3062	1192	547
