# Supplementary material for: Fine mapping and candidate gene analysis of Dravet syndrome modifier loci on mouse chromosomes 7 and 8
Source: Mamm Genome. 2024 Jun 11;35(3):334–45. doi: 10.1007/s00335-024-10046-3 (PMC11329421; doi:10.1007/s00335-024-10046-3)
Supplement: Supplementary file 4 — (PDF 115 KB) [file 335_2024_10046_MOESM4_ESM.pdf]

Supplementary Table S4. Effect of candidate gene coding variants determined by Ensembl Variant Effect Predictor (VEP).

SYMBOL	Variant ID	Location	Consequence	IMPACT	Transcript (canonical)	cDNA position	CDS position	Protein position	Amino acids	Codons	Ref Allele	Alt Allele	SIFT Prediction (Score)	PolyPhen-2 Prediction (Score)
Myod1	rs13472312	7:46026253-46026253	Missense	MODERATE	ENSMUST00000072514.3	356	157	53	M/V	Atg/Gtg	A	G	Tolerated (1)	Benign (0.000)
	rs13472315	7:46026292-46026292	missense	MODERATE	ENSMUST00000072514.3	395	196	66	P/S	Cct/Tct	C	T	Tolerated (0.21)	Probably Damaging (0.997)
	rs32790785	7:46027237-46027237	missense	MODERATE	ENSMUST00000072514.3	900	701	234	A/V	gCg/gTg	C	T	Tolerated (0.06)	Benign (0.001)
Sergef	rs31674298	7:46092740-46092740	missense	MODERATE	ENSMUST00000033127.12	1303	1268	423	D/A	gAc/gCc	T	G	Tolerated_low_confidence (1)	Benign (0.000)
	rs32795453	7:46092705-46092705	Missense	MODERATE	ENSMUST00000033127.12	1338	1303	435	R/G	Aga/Gga	T	C	Deleterious_low_confidence (0)	Benign (0.234)
Nav2	rs250301556	7:49058454-49058454	Inframe insertion	MODERATE	ENSMUST00000184945.8	673-674	576-577	192-193	-/QQQQ	-/CAGCAACAGCAA	-	CAGCAA CAGCAA	-	-
	rs31226051	7:49114575-49114575	missense	MODERATE	ENSMUST00000184945.8	2655	2558	853	D/G	gAc/gGc	A	G	Tolerated_low_confidence (1)	Benign (0.000)
	rs32337268	7:49197728-49197728	missense	MODERATE	ENSMUST00000184945.8	3453	3356	1119	T/M	aCg/aTg	C	T	Deleterious_low_confidence (0)	Possibly damaging (0.578)
	rs248206089	7:49197731-49197731	missense	MODERATE	ENSMUST00000184945.8	3456	3359	1120	V/A	gTc/gCc	T	C	Tolerated_low_confidence (1)	Benign (0.000)
Dbx1	rs246161289	7:49282284-49282290	Inframe deletion	MODERATE	ENSMUST00000032717.7	1048-1053	914-919	305-307	PAH/H	cCGGCGCac/cac	GCGCCG	-	-	-
Prmt3	rs32892158	7:49448109-49448109	missense	MODERATE	ENSMUST00000032715.13	949	818	273	V/A	gTt/gCt	T	C	Tolerated (1)	Benign (0.000)
Slc6a5	rs31048165	7:49561578-49561578	missense	MODERATE	ENSMUST00000056442.12	366	109	37	T/A	Acg/Gcg	A	G	Tolerated_low_confidence (1)	Benign (0.000)
Psd3	rs32698896	8:68573659-68573659	missense	MODERATE	ENSMUST00000212960.2	1131	521	174	G/E	gGg/gAg	C	T	Tolerated_low_confidence (0.17)	Benign (0.000)
	rs32882847	8:68573714-68573714	missense	MODERATE	ENSMUST00000212960.2	1076	466	156	R/G	Aga/Gga	T	C	Tolerated_low_confidence (1)	Benign (0.000)
Sh2d4a	rs36852848	8:68787743-68787743	missense	MODERATE	ENSMUST00000066594.4	1352	848	283	I/T	aTc/aCc	T	C	Deleterious (0.01)	Benign (0.000)
